# Arachnoid Cyst: A Sudden Deterioration

**DOI:** 10.7759/cureus.36552

**Published:** 2023-03-22

**Authors:** Khalid Y Fadul, Mohamed Ali, Amro Abdelrahman, Sara M I. Ahmed, Ameera Fadul, Hanna Ali, Mohamed Elgassim

**Affiliations:** 1 Emergency Medicine, Hamad Medical Corporation, Doha, QAT; 2 Emergency Medicine, Hamad General Hospital, Doha, QAT

**Keywords:** neuro-critical care, neuro-imaging, arachnoid cyst fenestration, status epilepticus, arachnoid cyst

## Abstract

Arachnoid cysts are relatively rare and usually asymptomatic. It can only be diagnosed through radiological imaging modalities. Some patients may develop symptoms such as seizures, headache, dizziness, or psychiatric symptoms.

We present a case of a 25-year-old male, previously healthy, who presented with sudden, repetitive episodes of seizure without regaining his consciousness. Computed tomography (CT) head scan showed a large cystic lesion that showed a rightward midline shift. Treatment was done surgically via endoscopic fenestration, and the patient remained symptom-free for one year. Most arachnoid cysts tend to remain asymptomatic throughout a patient's life span, allowing them to lead everyday normal lives; however, when these symptoms surface, they tend to be of a sudden nature requiring urgent surgical intervention. Our report follows the case of a young patient whose symptoms appear suddenly with triggers that led to status epilepticus. Our patient continued to suffer from multiple seizure attacks despite being on multiple anti-convulsive medications, and his symptoms eventually subsided via surgical intervention.

## Introduction

The first description of an arachnoid cyst (AC) in the medical literature was in the early 18th century by Bright et al. [1]. This rarely occurring neuronal anomaly encompasses what approximates to be 1% of all spontaneously occurring brain masses [2]. It can be traced back to both congenital and acquired pathophysiologies and is usually diagnosed earlier in life, with ages in literature ranging from as early as two years up to 40 years of age. Most of these diagnoses are made via incidental findings on neuroimaging [3]. Patients with AC can have various presentations, with the majority being asymptomatic and others having a full range of neurological symptoms ranging from mild headaches to cognitive impairment; seizures are commonly reported among AC patients, and reported symptoms also include vestibular symptoms [2].

The management line of such cysts tends to depend mainly on the symptomatology, with asymptomatic ones being managed conservatively and those with significant symptoms being managed surgically [4,5]. This report follows a symptomatic patient who eventually opted for a surgical option.

## Case presentation

A 25-year-old male previously healthy and with no family medical history of intracranial neoplasms or epilepsy was brought to the emergency department (ED) because of an attack or seizure. The family description of a mixture of a generalized repetitive jerky movement and muscle stiffening was typical of a generalized tonic-clonic seizure. While being transported by Emergency Medical Services (EMS), the patient experienced two additional attacks of similar nature. The patient didn't regain consciousness between the seizure episodes.

In the ED, he was hemodynamically stable, and physical examination was unremarkable except for equal but non-reactive dilated pupils and a Glasgow Coma Scale (GCS) of 6 (E1V1M4), so the airway was immediately secured by endotracheal intubation. The patient required multiple anti-convulsive agents (Midazolam, Propofol, and Levetiracetam) to control his seizures. Laboratory workup was unremarkable, including normal thyroid stimulating hormone (TSH).

**Figure 1:**
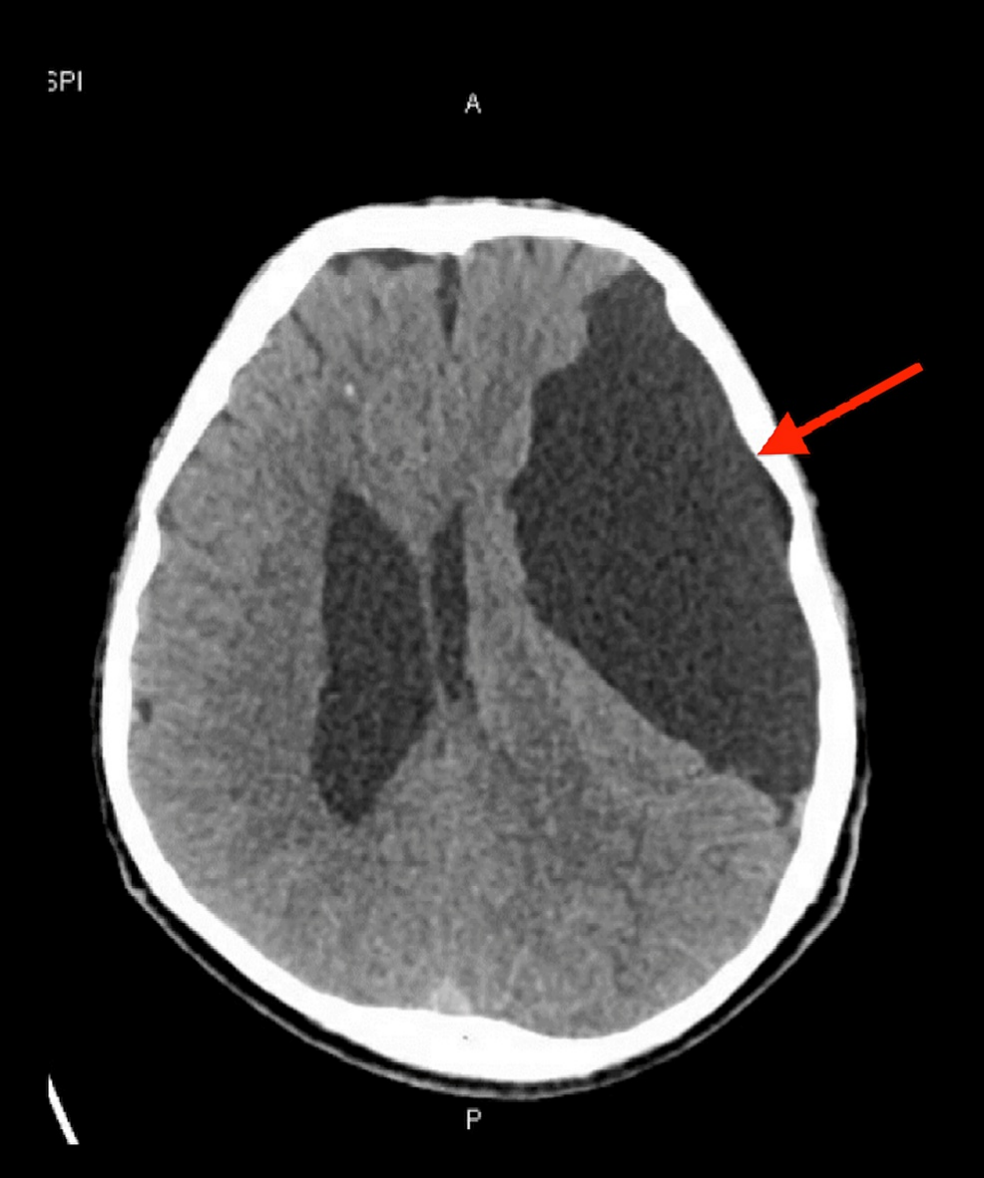
Initial CT head scan showing a large CSF density extra-axial cystic lesion along the left cerebral convexity consistent with an arachnoid cyst, causing a mass effect and midline shift to the right CT: computed tomography; CSF: cerebrospinal fluid

**Figure 2:**
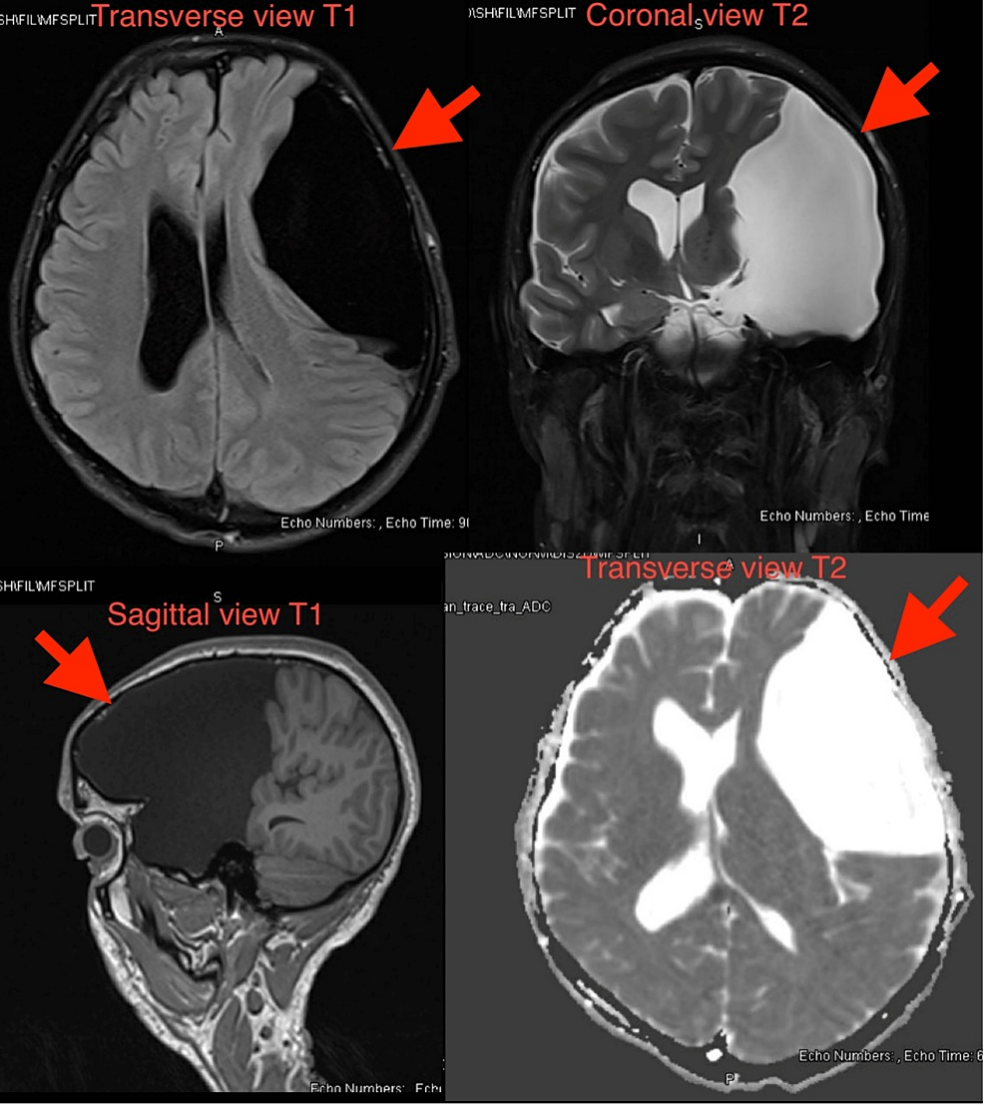
Multiple cuts from MRI head showing 6.5 x 11.5 x 12.5 cm left hemispheric arachnoid cyst with an 8.5 mm midline shift to the right MRI: magnetic resonance imaging

An urgent plain computed tomography (CT) head scan showed a large cerebrospinal fluid (CSF) density extra-axial cystic lesion along the left cerebral convexity consistent with an AC, causing a mass effect and midline shift to the right (Figure 1). Non-contrast magnetic resonance imaging (MRI) confirmed a 6.5 x 11.5 x 12.5 cm left hemispheric AC with an 8.5 mm midline shift to the right (Figure 2).

The Neurosurgery on-call team was consulted and they admitted the patient to the surgical intensive care unit (SICU) for observation and consulted neurology for their input on convulsions control; Neurology did electroencephalogram (EEG) showing low-frequency high amplitude delta activity with occasional 1-2 second periods of suppression with additional nearly continuous theta activity over the right anterior quadrant.

**Figure 3:**
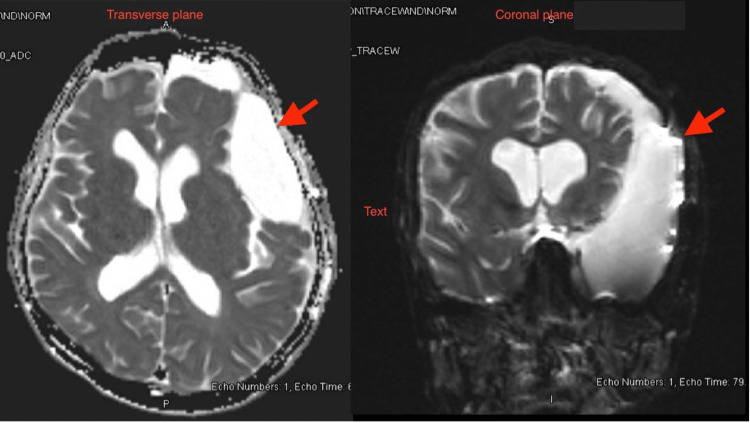
Follow-up CT head scan was done after one week from arachnoid cyst fenestration showing a decrease in the size CT: computed tomography

Two days following the admission, he underwent a craniotomy and fenestration of the AC. The patient had another episode of convulsion the next day after intensivists attempted to wean them off the sedation controlled by Levetiracetam. A follow-up CT scan done a week later showed a reduction in the size of the AC and regression of the midline shift, and during this period, the patient remained seizure-free (Figure 3).

The patient was successfully weaned off sedation and extubated on day 4 post-operation; he was eventually discharged on Levetiracetam with full neurological function with advice for follow-up at the Neurology and Neurosurgery clinics. One year follow-up showed only a single seizure episode after discharge, self-resolved and short-lived, reported by his family. The patient didn't complain of any neurological symptoms during this period.

## Discussion

AC are developmental cysts of non-malignant nature containing colorless fluid resembling CSF. It may develop in the arachnoid membrane and subarachnoid space throughout the central nervous system [6]. Although an identifiable underlying cause for the formation of AC is yet to be identified. It has been suggested by Helland et al. that there is a possibility of a genetic element in the formation of AC [7]. It has also been proposed that in the early stages of the formation of AC, there is communication between the cyst and the subarachnoid space, evidenced by radioisotopes' entry into the cyst [6]; Al-Holou et al. reported the most common site for AC is the middle cranial fossa and that males are more susceptible to AC than females [8]. In contrast, in our patient, the cyst was located in the frontotemporal area.

Most cysts are discovered incidentally through radiological imaging (CT or MRI), and the majority are asymptomatic. Symptomatic presentations may occur, including convulsions, abnormal behavior, and cognitive impairment [9]. Endocrinopathies also have been reported [10]; our patient presented with status epilepticus without any evidence of visual disturbance or endocrinopathy; his family denied any abnormal behavior prior to the seizures.

Regarding the management of patients diagnosed incidentally, it is preferred to observe using serial MRIs, endocrinological tests, and a comprehensive ophthalmologic assessment and treated as per significant findings. As for the patients with symptoms, the treatment is surgical via cyst fenestration either by endoscopy or craniotomy [11]; surgical intervention should be reserved for those AC in which their size impacts their surroundings, whether it be via compression of neural structures, formations of hydrocephalus or symptomatic refractory presentations [4]. However, it has been reported that some patients were managed with medical treatment without the need to resort to surgical intervention [10]; our patient presented with frequent convulsions with CT finding of a large AC on imaging, so he was treated surgically via craniotomy due to the mass effect of the AC.

## Conclusions

AC can present with a variety of symptoms or can be asymptomatic. Diagnosis with adequate neuro-imaging helps with planning appropriate therapeutic approach. Invasive approach is usually reserved for cysts causing neurological symptoms due to compression to adjacent structures, hydrocephalus, or refractory symptoms.
